# The Southeast Alaska Tribal Ocean Research (SEATOR) Partnership: Addressing Data Gaps in Harmful Algal Bloom Monitoring and Shellfish Safety in Southeast Alaska

**DOI:** 10.3390/toxins12060407

**Published:** 2020-06-19

**Authors:** John R. Harley, Kari Lanphier, Esther G. Kennedy, Tod A. Leighfield, Allison Bidlack, Matthew O. Gribble, Christopher Whitehead

**Affiliations:** 1 Alaska Coastal Rainforest Center, University of Alaska Southeast, Juneau, AK 99801, USA; albidlack@alaska.edu; 2 Sitka Tribe of Alaska, Sitka, AK 99835, USA; kari.lanphier@sitkatribe-nsn.gov (K.L.); egkennedy@ucdavis.edu (E.G.K.); chris.whitehead@sitkatribe-nsn.gov (C.W.); 3 National Oceanic and Atmospheric Administration, National Centers for Coastal Ocean Science, Charleston, SC 29412, USA; tod.leighfield@noaa.gov; 4 Gangarosa Department of Environmental Health, Rollins School of Public Health, Emory University, Atlanta, GA 30322, USA; matt.gribble@emory.edu

**Keywords:** environmental health, oceans and seas, paralytic shellfish poisoning, prevention, primary, community-based participatory research

## Abstract

Many communities in Southeast Alaska harvest shellfish such as mussels and clams as an important part of a subsistence or traditional diet. Harmful algal blooms (HABs) of phytoplankton such as *Alexandrium* spp. produce toxins that can accumulate in shellfish tissues to concentrations that can pose a hazard for human health. Since 2013, several tribal governments and communities have pooled resources to form the Southeast Alaska Tribal Ocean Research (SEATOR) network, with the goal of minimizing risks to seafood harvest and enhancing food security. SEATOR monitors toxin concentrations in shellfish and collects and consolidates data on environmental variables that may be important predictors of toxin levels such as sea surface temperature and salinity. Data from SEATOR are publicly available and are encouraged to be used for the development and testing of predictive algorithms that could improve seafood risk assessment in Southeast Alaska. To date, more than 1700 shellfish samples have been analyzed for paralytic shellfish toxins (PSTs) in more than 20 locations, with potentially lethal concentrations observed in blue mussels (*Mytilus trossulus*) and butter clams (*Saxidomus gigantea*). Concentrations of PSTs exhibit seasonality in some species, and observations of *Alexandrium* are correlated to sea surface temperature and salinity; however, concentrations above the threshold of concern have been found in all months, and substantial variation in concentrations of PSTs remain unexplained.

## 1. Introduction

Harmful algal blooms (HABs) pose a threat to coastal communities, especially those dependent on marine resources such as fish and shellfish [1,2,3]. Toxins produced by eukaryotic phytoplankton and cyanobacteria can accumulate in significant quantities in some species and cause illness or even death in humans and wildlife [4]. There is strong evidence that warming ocean temperatures due to climate change will expand the geographic range and bloom season of several HAB species, which makes monitoring and responding to HABs a priority for coastal community health [5,6].

Blooms of the dinoflagellate *Alexandrium* spp. (henceforth *Alexandrium*) are particularly concerning due to their production of a suite of toxins known as paralytic shellfish toxins (PSTs) [7]. PST is the name given to a group of several compounds and congeners (e.g., saxitoxin) that are potent neurotoxicants. At sufficient concentrations PSTs can cause paralytic shellfish poisoning (PSP). Saxitoxin was in fact named after the Alaskan butter clam (*Saxidomus gigantea*) from which it was first isolated [8]. The earliest documented outbreak of PSP occurred in Alaska in 1799 [9], and since then dozens of outbreaks have been reported in Alaska, British Columbia, and Washington resulting in hospitalizations and deaths [10,11,12].

**Figure 1:**
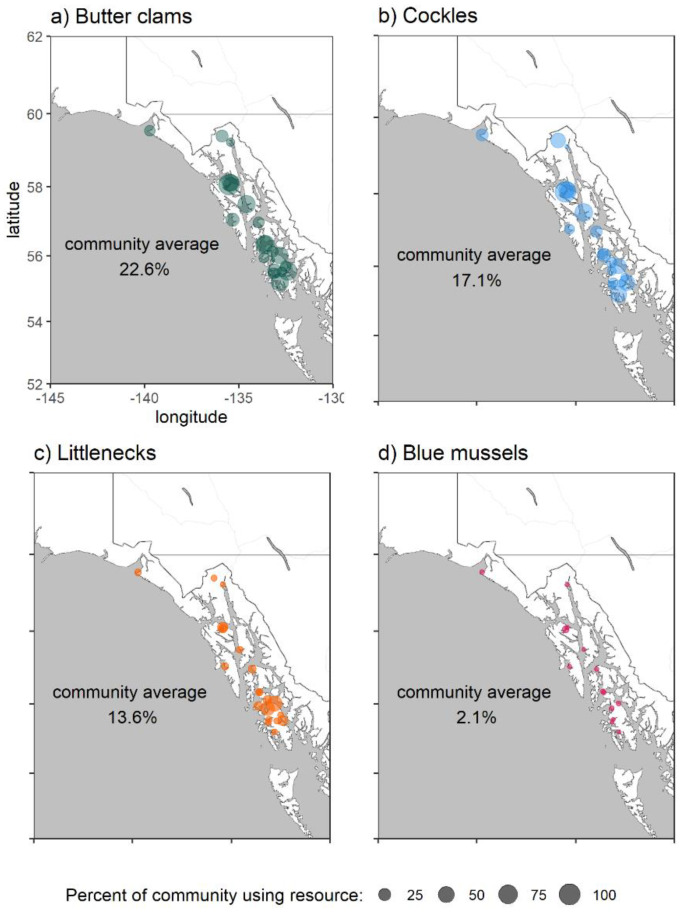
Subsistence use of bivalves in Southeast Alaska by community including (**a**) butter clams (*S. gigantea*), (**b**) cockles (*C*. *nuttallii*), (**c**) littlenecks (*L. staminea*) and (**d**) blue mussels (*M. trossulus*). The size of each circle is proportional to the percentage of households using that resource in each community. The community average is weighted using the size of each community. Data are from the Alaska Department of Fish and Game (ADF&G) Community Subsistence Information System (CSIS) [15] aggregated from multiple surveys (1984–2014).

**Figure 2:**
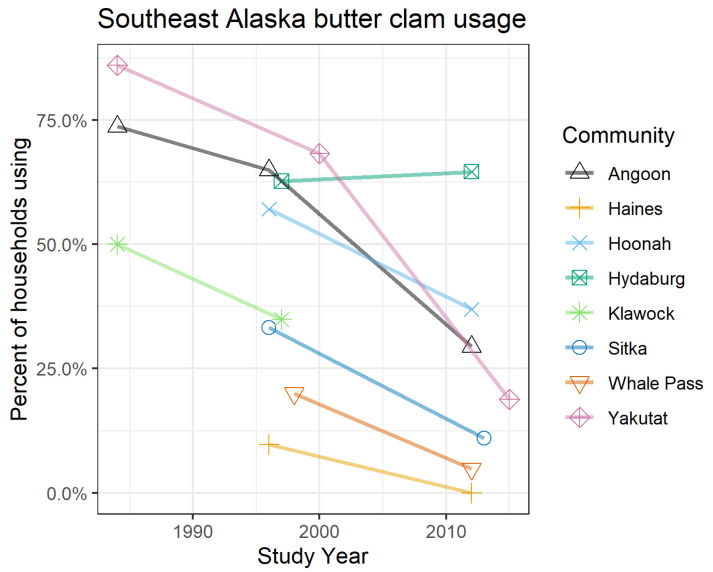
Surveyed usage of butter clams (*S. gigantea*) has declined in several communities in Southeast Alaska. Respondents stated concerns about paralytic shellfish poisoning (PSP) among other reasons for harvesting fewer marine invertebrates [14]. Data are from the Community Subsistence Information System (CSIS) [15].

Shellfish are an important cultural and subsistence item for many Alaska Native communities and coastal populations [13]. Subsistence data collected by the Alaska Department of Fish and Game show that shellfish resources are an important resource for many communities in Southeast Alaska (Figure 1), although the use of some of these organisms, especially butter clams, appears to be declining in some communities (Figure 2) [14,15]. Respondents to subsistence surveys reported concerns over PSP as a contributing factor to decreased utilization of shellfish [14].

**Figure 3:**
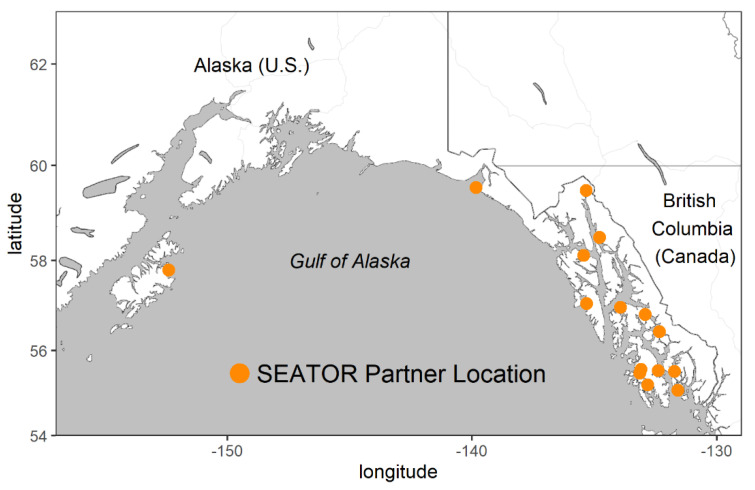
Map of Southeast Alaska Tribal Ocean Research (SEATOR) shellfish and phytoplankton sampling locations (as of March 2020) in Alaska. Axes are presented in decimal degrees.

Despite concerns over the safety of shellfish, butter clams, Pacific blue mussels (*Mytilus trossulus*, part of the *M. edulis* complex) and cockles (*Clinocardium nuttallii*) have been and continue to be traditional and subsistence diet items for communities in Southeast Alaska [16]. Identifying when and where shellfish may be unsafe to consume is a major concern for Alaska Native communities. Unlike other states (e.g., Washington, Oregon) that have state-sponsored shellfish monitoring, there is currently no regular monitoring program for shellfish toxins provided by state organizations within Alaska. In 2013, the Sitka Tribe of Alaska (STA) partnered with several other Southeast tribal organizations (a full list of partners is presented in the acknowledgements) to form the Southeast Alaska Tribal Toxins network (SEATT), with the goal of gathering data on shellfish toxins (including PSTs) and dynamics of HAB species in Southeast Alaska. In 2014, the network was absorbed into a broader monitoring program called the Southeast Alaska Tribal Ocean Research (SEATOR) network (Figure 3). In addition to monitoring shellfish toxins, SEATOR also addresses data gaps in our understanding of HAB dynamics in Southeast Alaska. SEATOR partners collect observations of phytoplankton including harmful algal species (*Alexandrium*, *Pseudo-nitzschia* spp., *Dinophysis* spp.) and environmental variables such as sea surface temperature (SST) and salinity. It is important to note that SEATOR itself does not open or close beaches; rather, they issue consumption advisories and allow tribal communities and partners to make their own decisions and recommendations based on available data.

Tribal governments, recreational harvesters, subsistence harvesters and researchers can access shellfish toxicity data through the SEATOR website and use this information to make shellfish harvest recommendations for themselves or their communities. Real time and near real time observations of HABs and PST concentrations are essential for informed shellfish aquaculture, subsistence, and recreational harvest, but there has also been a motivation among the scientific community and stakeholders to leverage concurrent environmental datasets to determine drivers of HABs [17,18]. Studies examining environmental drivers of HABs and PSTs have demonstrated that SST, salinity and wind-driven mixing are important drivers of *Alexandrium* bloom formation and PST production [17,19,20,21]. Many of these studies have relied on high-resolution datasets generated by various federal or state agencies (e.g., National Oceanic and Atmospheric Administration (NOAA), National Aeronautics and Space Administration (NASA)) combined with regional shellfish toxicity monitoring data. However, the remoteness, extreme tidal currents and complex geography of the Southeast Alaska coastline present numerous hurdles to the production of ocean data products such as buoy-based observations. Even well-distributed networks of monitoring equipment might miss intricacies of physical and chemical dynamics on small scales [22]; thus, sampling of local conditions (i.e., SST, salinity, air temperature) near or at shellfish and plankton sampling locations is a valuable addition to studies of shellfish toxins and HAB dynamics.

The purpose of this paper is to highlight some of the data generated from the SEATOR network regarding PST concentrations and *Alexandrium* observations in Southeast Alaska. We discuss species-specific profiles of PSTs and the role of temperature and salinity on *Alexandrium* in more than a dozen locations in Southeast Alaska. We also highlight the need for continued monitoring of environmental health end points (i.e., PST concentrations) in conjunction with environmental models of HAB dynamics and forecasts.

## 2. Results and Discussion

### 2.1. HAB and Oceanographic Observations

**Figure 4:**
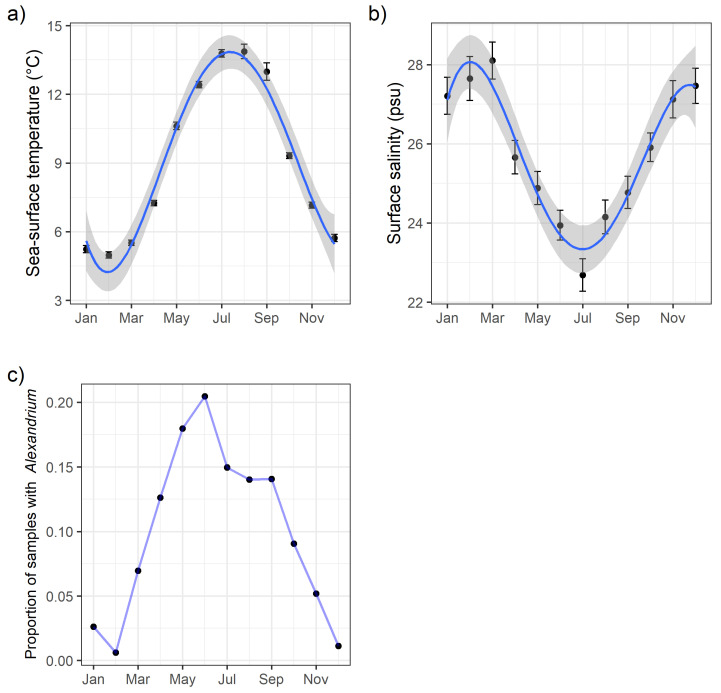
(**a**) Sea surface temperatures and (**b**) salinity data collected by SEATOR partners from 2016 to 2019. Error bars represent standard error for each month, and the trend line is a quartic spline interpolation. These variables were measured in conjunction with phytoplankton observations. The proportion of net tows for each month that contained *Alexandrium* is presented in panel (**c**). In this plot, abundance estimates were grouped as presence/absence; thus, samples coded as Present, Common, and Bloom were considered samples with *Alexandrium*.

The regional SEATOR partnership has developed a sampling protocol that has produced a valuable longitudinal dataset of HAB observations since 2016. The oceanographic and phytoplankton data that have been recorded represent some of the most extensive observation efforts in many of these communities. Using hand-collected surface water samples, nearly 2400 observations of salinity (refractometer) and temperature (thermometer) have been recorded by SEATOR partners (Figure 4). Although there are some marine weather stations that record and stream SST data in near real time, the spatial distribution of these observations might miss more localized effects of freshwater input or tidal flux, which could affect both SST and salinity. To our knowledge there are only a few currently operating stations generating publicly available salinity data in Southeast Alaska. Both SST and salinity have been shown to be key variables in predicting blooms of *Alexandrium* [17,19,23]; thus, the collection and distribution of these data in near real time is essential for model-building and forecasting. There are additional variables that would be informative and valuable to assess on this regional scale, including nutrient dynamics and molecular ecology, but with limited funding and resources the SEATOR network has focused on data that can be collected relatively inexpensively and rapidly, facilitating a real-time, responsive network.

**Figure 5:**
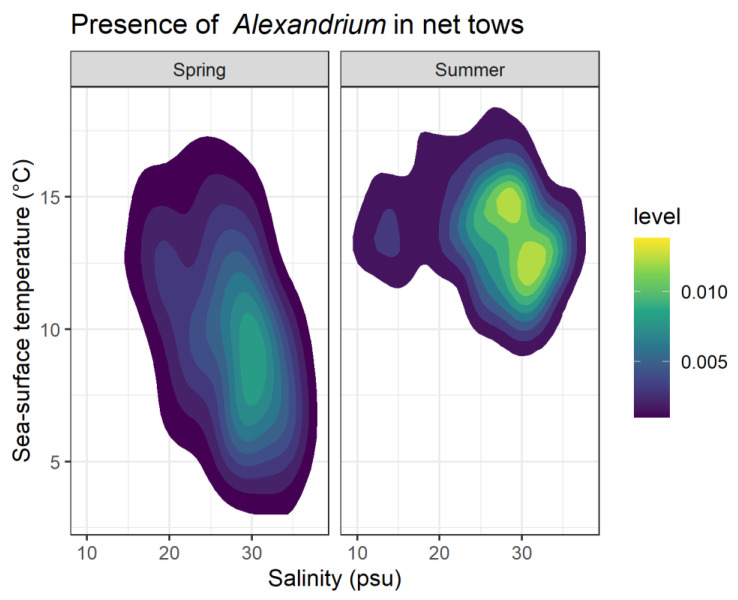
Two-dimensional kernel density estimation of *Alexandrium* presence in phytoplankton net tows in relationship to measured salinity and SST. Color gradient represents density of *Alexandrium* observations (low = blue, yellow = high) during spring (April–June) and summer (July–September). Plotted here are 1176 observations of *Alexandrium* identified using microscopy by SEATOR partners. The color of each polygon corresponds to the density of observations within that kernel (level).

SST in particular has been shown to be a key driver of *Alexandrium* blooms. Bill et al. [23] showed optimal growth for Salish Sea isolates of *Alexandrium* at temperatures between 10 and 24 °C, which are typically observed in Southeast Alaska from June to September (Figure 4a and Figure 5). The majority of observations of *Alexandrium* in Southeast Alaska (63%) occurred in water temperatures >10 °C; however, in the spring *Alexandrium* is often seen in water temperatures 8 °C or colder (Figure 5).

Salinity in Southeast Alaska also exhibits a seasonal cycle (Figure 4b), with maximum salinity occurring in midwinter due to decreased freshwater input from glacial melt and precipitation generally falling in the form of snow [24]. However, salinity in the upper surface water (1 m), where SEATOR partner sampling typically occurs, can be variable, especially during heavy precipitation events [25]. Bill et al. [23] described *Alexandrium* as tolerant to a wide range of salinities, and although Southeast Alaska is not as saline as other regions where *Alexandrium* is observed, we also found cells across a wide range of salinities.

While historically PSTs have been the main shellfish toxins of concern, it is important to note that other toxin-producing HAB species are monitored by SEATOR and have been observed in Southeast Alaska. In particular, *Pseudo-nitzschia* spp., which can produce domoic acid causing amnesic shellfish poisoning, and *Dinophysis* spp., which can produce okadaic acid and its congeners causing diarrhetic shellfish poisoning, have been observed on 912 (27.5% of samples) and 348 (10.5% of samples) occasions respectively since 2015 (data not shown). High concentrations of domoic acid have disrupted shellfisheries in California, Oregon and Washington, and extremely large blooms such as one observed in 2015 can have large-scale ecosystem impacts [26]. Warming ocean conditions, especially anomalously warm conditions, could lead to the proliferation of HAB species that have not been historically problematic for Alaska [5].

### 2.2. PSTs in Southeast Alaska Shellfish

Since the SEATOR network began monitoring shellfish toxins in Southeast Alaska, the Sitka Tribe of Alaska Environmental Research Laboratory (STAERL) has received and analyzed more than 1700 shellfish samples from tribal partners, commercial harvesters and recreational harvesters. Concentrations of PSTs are highly variable both within and between species, and maximum concentrations have exceeded the FDA threshold (80 micrograms STX equivalents per 100 g shellfish tissue) in all consistently monitored species (blue mussel, butter clam, cockle, littleneck clam, Table 1).

**Figure 6:**
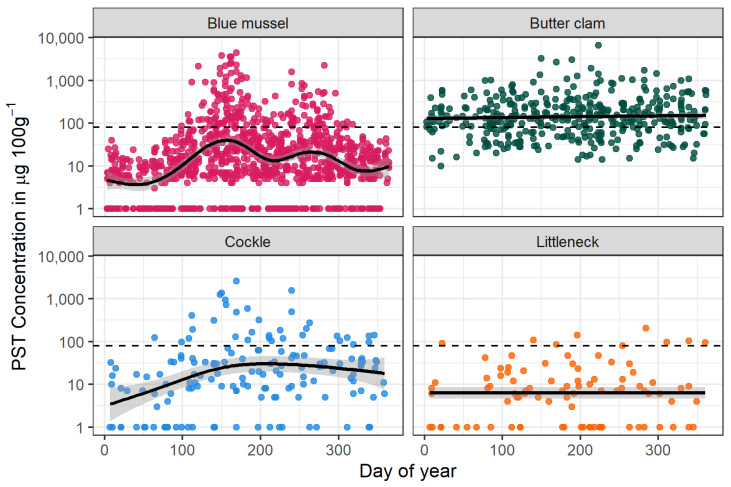
PST concentrations in select species of bivalves from Southeast Alaska 2015–2019 measured by receptor-binding assay (RBA) analysis. Note that values are displayed on a log axis due to the orders of magnitude of variability in seasonal PST concentrations in some species. The dashed line represents the FDA threshold of 80 µg 100 g^−1^. Trend lines are generalized additive model (GAM) smoothing curves, presented with confidence interval (shaded region).

Similar to what has been described in other regions and in laboratory conditions (see [27]), we found that some species (i.e., blue mussels) exhibited a strong seasonal pattern of toxicity at many sampling locations, where peak toxicities occurred in May–June during spring blooms and often had a smaller peak in late August or September (Figure 6). Blue mussels (*M. edulis*) have been shown to have fast uptake and depuration rates, making them an ideal sentinel species for management recommendations and modeling [27,28].

In contrast to fast detoxifiers like the blue mussel, the butter clam has been known to retain PSTs for extended time periods (months to years) [27,29]. Several hypotheses have been proposed to explain low depuration rates, including selection pressure favoring retention of PSTs as a chemical defense for the butter clam against predation [29]. While detoxification kinetics are poorly understood among bivalves, it has been suggested in this and other species that environmental variables such as temperature, salinity and the availability of nontoxic algae might influence detoxification rates [27]. Therefore, having access to environmental data is important not only for forecasting HAB events but potentially also for predicting detoxification rates in bivalve species.

A common misconception about shellfish harvest is that shellfish are safe to consume only in months that have an “r” in the name (September–April). In fact, incidents of PSP have occurred in Alaska in every month [10,11], and our data show that PST concentrations have exceeded the FDA threshold for safe consumption throughout the year. Large blooms of *Alexandrium* have been seen in April and October in some communities, resulting in mussel concentrations of PST > 80 µg 100 g^−1^. Butter clams, which can retain PSTs for more than a year, have tested above the FDA threshold in every month in some communities. It is important to note that there is significant spatial variation in PST concentrations. While most communities have seen elevated PST concentrations at some point since monitoring began in 2016, other communities have seen infrequent or no mussel samples above the FDA threshold. These observations demonstrate the importance of recording and analyzing environmental variables, which can potentially explain some of the variation in seasonal PST concentrations.

### 2.3. Value of These Data as a Platform for Modeling Efforts

Understanding environmental drivers of HABs is critical for developing accurate forecasts and providing shellfish harvesters and stakeholders with data to make informed decisions. Blooms of HAB species, including *Alexandrium*, respond to different environmental forcings in different regions—thus the first steps toward generating forecasts are gathering of baseline data regarding algal species presence/absence, gathering shellfish toxicity data and assessing the availability of near real-time data sources.

Moore et al. [19] and Finnis et al. [17] incorporated historical environmental variables from Puget Sound (Washington, WA, USA) and the Vancouver Island area (British Columbia, Canada) to examine drivers of PST. While there were site differences within each region, the important drivers of PSTs were generally SST, salinity, air temperature and freshwater discharge. As discussed in Brown et al. [30], there is a technical and scientific hurdle between modeling historical blooms and forecasting blooms in real time, even though some models effectively hindcast blooms using machine learning [17]. With the exception of salinity, many of the variables that have been described as important drivers of *Alexandrium* blooms in the Eastern Pacific (Washington, British Columbia, Alaska) are collected at numerous stations in Southeast Alaska and across the state in near real time and are available to the public and aggregated through the U.S. Integrated Ocean Observing System’s Alaska Ocean Observing System (AOOS) portal [31].

While there is sufficient access to historical and streaming environmental data to undertake modeling environmental drivers of HABs in Southeast Alaska, we stress that thorough quality control and understanding of relevant physical dynamics of the region are essential [32]. The SEATOR network has developed a widespread and multifaceted monitoring system, yet the paucity of data before the network’s inception will be a hurdle in modeling events such as HABs that have significant spatial and temporal variation.

## 3. Conclusions

SEATOR is responsive to community concerns and generates and consolidates high-quality data that can be complemented by analytic tools to improve environmental health in communities in Alaska that rely on subsistence harvest of shellfish. While there are numerous hurdles to studying and modeling complex environmental phenomena such as HABs in Southeast Alaska, efforts in recent years to concatenate streaming environmental sensors as well as environmental health end points (PST concentrations) have created publicly available data sets that can be accessed and utilized by researchers, stakeholders and managers. Future efforts examining different factors of HAB formation and toxin production (i.e., grazer abundance, nutrient concentrations, molecular genotyping) can benefit from the longitudinal data collected by the SEATOR network. The continued collection and curation of these data is essential for future modeling efforts and developing operational forecasts for HABs and shellfish toxins.

## 4. Materials and Methods

Shellfish samples were collected biweekly from SEATOR partner locations (Figure 3) and shipped whole on ice to the STAERL for analysis. For blue mussels, a variable number of individuals (20–100) were harvested and shucked until 100 g of tissue was obtained. For other larger species, a minimum of six individuals were harvested, shucked and homogenized.

Concentrations of PSTs were determined using the receptor-binding assay (RBA) based on Van Dolah et al. [33] and the AOAC Official Method 2011.27. Briefly, five grams homogenate was placed in a sample tube with 5 mL of 0.1 M HCl and placed in a beaker of boiling water on a hot plate for 5 min with caps loosened. The resulting mixture was cooled, adjusted with 5 M HCl to pH 2–3 and then centrifuged at 3000× *g* for 20 min.

To prepare the membranes, 1.25 mL of frozen porcine brain membrane homogenate (Millipore Sigma, Saint Louis, MO, USA) was thawed and vortexed with 10.75 mL of 3-(*N*-Morpholino) propane sulfonic acid (MOPS). Plate wells were pre-wet with 35 μL MOPS, to which we added 35 μL of standard, sample supernatant, or quality control (QC), 35 μL of prepared [H3] STX (10–15 nM in MOPS, pH 7.4, American Radiolabeled Chemicals, St. Louis, MO, USA) and 105 μL membrane preparation. Plates were then covered and incubated at 4 °C for 1 h.

Following incubation, plates were filtered using a multiscreen vacuum manifold (Millipore Sigma, St. Louis, MO, USA), and each well was rinsed twice with 200 μL MOPS. Fifty microliters of scintillation cocktail (OptiPhase, PerkinElmer, Waltham, MA, USA) was added to each well. After 30 min, trays were read using a microplate counter (MicroBeta2, PerkinElmer, Waltham, MA, USA). Samples were all run in triplicate, and samples were analyzed using at least two different dilutions on an eight-point standard curve. Two QC runs (3 nM [H3] STX) were included with each plate. All replicates were averaged provided they fell within the standard curve and concentrations from different dilutions fell within an acceptable range.

Several species of shellfish were analyzed from SEATOR partners including eastern softshell clams (*Mya arenaria*, n = 14), horse clams (*Tressus capax*, n = 9), surf clams (*Mactromeris polynyma*, n = 5) and California mussels (*Mytilus californianus*, n = 4); however, the most common species for monitoring and consumption were blue mussels (*M. trossulus,* n = 1015), butter clams (*S. gigantea,* n = 364), cockles (*C*. *nuttallii*, n = 149) and littleneck clams (*Leukoma staminea*, n = 88). PST concentrations were uploaded to the SEATOR website (seator.org), typically within 48 h of collection.

SEATOR partners also conducted weekly or biweekly phytoplankton monitoring using methods developed by the SoundToxins Project [34]. Three-minute net tows were conducted with a 20-μm plankton net, and phytoplankton were identified using light microscopy (usually 10× to 40× magnification). Small subsamples of concentrated phytoplankton were analyzed on a ruled slide (64 2-mm squares). Partners attempted to identify all phytoplankton at least to genus, with particular attention paid to harmful algal species including *Alexandrium* spp., *Pseudo-nitzchia* spp., and *Dinophysis* spp. Species identification of some HAB species can be extremely difficult, potentially requiring molecular techniques [7]; thus, partners only identified *Alexandrium* to genus. Harmful algal species were assigned a relative abundance along an ordinal scale of Absent, Present, Common, and Bloom, although since flow rate was not precisely calculated during net tows, these designations are not directly comparable to outright in-water concentrations. Instead, relative abundances were described adapting the methods developed and used by programs in Washington state (SoundToxins and the Olympic Regional HAB group) that provide a qualitative estimate of abundance [34,35]. In these protocols, relative abundances are not rigidly defined in terms of cell counts but are an estimate from the observer and are species-specific. The goal of these qualitative assessments was to create easily interpretable action thresholds for communities and harvesters rather than provide absolute concentrations [35]. In general, species that were Present were either a single cell or a few cells per slide, species that were Common were present in most squares of the slide, and species labeled as Bloom generally had one or more cells per square. However, we reiterate that these indices are species-specific, so designations were normalized to particular species; for example, a Bloom designation for *Alexandrium* might be assigned for 20 cells per slide since *Alexandrium* is very rarely seen at concentrations of more than a few cells, while a designation of Bloom for *Pseudo-nitzchia* might indicate >20 cells per grid square since *Pseudo-nitzchia* is often seen at moderate concentrations. Here we present data as Presence/Absence (i.e., Figure 4c and Figure 5) by coding Present, Common and Bloom as Present, and to facilitate comparisons with absolute concentrations or other abundance estimates we suggest others do the same.

During net tows, partners also collected SST and air temperature using a digital thermometer and salinity using a handheld refractometer. Phytoplankton, temperature and salinity observations were uploaded into the SoundToxins database and made available to the public through the SEATOR data portal. Although the availability of these data were limited by the frequency of observations and the expediency with which the partners entered their observations, data were typically available through SEATOR the same day observations were made.

Subsistence harvest data (Figure 1 and Figure 2) were collected by the ADF&G Division of Subsistence aggregated across multiple years of household surveys. Aggregated data were accessed through the Alaska CSIS (accessed January 2020) and used with permission here.

## Figures and Tables

**Table 1:** Maximum PST concentration (μg 100 g^−1^) in select species (2016–2019) measured by the Sitka Tribe of Alaska Environmental Research Laboratory (STAERL). Shellfish samples are from SEATOR partner sites (see Figure 3).

Species	Year			
	2016	2017	2018	2019
Blue mussel	**916**	**3791**	**2243**	**4412**
(*M. trossulus*)				
Butter clam	**723**	**6624**	**1712**	**3081**
(*S. gigantea*)				
California mussel	NT	NT	24	29
(*Mytilus californianus*)				
Cockle	**202**	**1367**	**1565**	**2603**
(*C. nuttallii*)				
Eastern softshell	15	6	8	21
(*Mya arenaria*)				
Horse clam	13	BDL	42	37
(*Tresus capax*)				
Littleneck clam	24	47	**206**	**142**
(*Leukoma staminea*)				

Note: Values in bold are above the FDA threshold for human consumption (80 μg 100 g^−1^). NT = Not tested. BDL = Below detection level.
